# Dietary Intake and Its Relationship to Different Body Mass Index Categories: A Population-Based Study

**Published:** 2018-09-08

**Authors:** Ali Asghar Rashidi, Ali Reza Heidari Bakavoli, Amir Avan, Malihe Aghasizade, Hamideh Ghazizadeh, Maryam Tayefi, Sayyed Saeid Khayyatzadeh, Mahmoud Ebrahimi, Mohsen Moohebati, Mohammad Safarian, Mohsen Nematy, Mostafa Sadr-Bazzaz, Gordon A. Ferns, Majid Ghayour Mobarhan

**Affiliations:** ^1^ Department of Nutrition, School of Medicine, Mashhad University of Medical Sciences, Mashhad, Iran; ^2^ Cardiovascular Research Center, School of Medicine, Mashhad University of Medical Sciences, Mashhad, Iran; ^3^ Metabolic Syndrome Research Center, Mashhad University of Medical Sciences, Mashhad, Iran; ^4^ Department of Modern Sciences and Technologies, School of Medicine, Mashhad University of Medical Sciences, Mashhad, Iran; ^5^ Clinical Research Unit, Mashhad University of Medical Sciences, Mashhad, Iran; ^6^ Nutrition and Food Security Research Center, Shahid Sadoughi University of Medical Sciences, Yazd, Iran; ^7^ Department of Nutrition, Faculty of Health, Shahid Sadoughi University of Medical Sciences, Yazd, Iran; ^8^ Brighton & Sussex Medical School, Division of Medical Education, Flamer, Brighton, UK

**Keywords:** Dietary intake, Body mass index, Nutrient, Obesity

## Abstract

**Background:** Obesity is a major public health problem because of its associated diabetes mellitus and cardiovascular disease. We aimed to explore the relationship between dietary macronutrients and adiposity in a cohort study, representative of the city of Mashhad in northeastern Iran.

**Study design:** A cross-sectional study.

**Methods:** The population sample (9847) derived from Mashhad stroke and heart atherosclerotic disorders (MASHAD: 2010-2020) and was obtained using a stratified-cluster method. The subjects were separated into 4 groups by body mass index status: normal weight, underweight, overweight and obese individuals. Individuals with mean age of 48.33 ±8.26 yr were recruited and anthropometric and biochemical factors were measured in all the subjects. Individual dietary intakes were assessed using 24-h dietary recall Dietplan6. Univariate and multivariate analyses were conducted before and after adjustment for age, gender and energy intake.

**Results:** Obese individuals were significantly less physically active. They had higher levels of serum fasted lipid profile, hs-CRP, uric acid, and glucose, and blood pressures compared to normal weight individuals (*P*=0.001). There was a significant difference in the dietary intakes of the groups categorized by obese before adjustment for energy intake in the obese compared to the normal weight group. These differences remained statistically significant for Trans fatty acid (*P*=0.033), lactose (*P*=0.009), fructose (*P*=0.025), glucose (*P*=0.017), sucrose (*P*=0.021) and maltose (*P*=0.015) after adjustment for energy intake.

**Conclusion:** Our findings demonstrate a significant association between dietary Trans fatty acid and total sugar intake with adiposity in a representative population sample from northeastern Iran.

## Introduction

Obesity is increasing globally and associated with several other co-morbidities, including diabetes mellitus and cardiovascular disease. These latter associations may be attributable in part to the higher prevalence of micronutrient deficiencies in obese people is higher compared to normal weight individuals^1-4^, whilst weight gain is due to an imbalance between energy intake and expenditure^5^. It is not clear whether weight gain is related to the macronutrient source of the increased energy intake, or merely related to the total energy consumption from whichever source. Obesity may be reduced by reducing dietary fat^6^ although this is not a consistent finding^7,8^.

Because of enormous public health impact of obesity, identifying the dietary factors associated with its causation is important if the global trend for increasing diabetes and cardiovascular disease are to be contained. Moreover, whilst there is a high prevalence of obesity in the Iranian population, the relationship between the macronutrient intake and obesity has not been extensively studied in this population.

We aimed to explore the relationship between dietary macronutrients and adiposity in a cohort study, representative of the city of Mashhad in northeastern Iran.

## Methods

### Study Population

The population sample derived from Mashhad stroke and heart atherosclerotic disorders (MASHAD: 2010-2020) and was obtained using a stratified-cluster method. The study design, sample selection, characteristics of study participants as well as details on data collection methods have been published^9^. Demographic information such as age, education level, marriage status, current smoking and job status was obtained by face to face interview^9,10^. The subjects (n=9809) were of mean age of 48.33±8.26 year. Pregnant and breastfeeding women, patients who had systemic disease, and patients taking any drug (including lipid-lowering drugs) were excluded from the study. They also had no known history of infectious diseases, a family history of stroke, myocardial infarction, and diabetes mellitus.

Informed consent was obtained from all participants using protocols approved by the Ethics Committee of the Mashhad University of Medical Sciences, Mashhad, Iran.

### Anthropometric and Biochemical Measurements

Anthropometric parameters including body weight, height, waist and hip circumference were measured using a standard protocol. Body mass index (BMI) was calculated as weight (kg) divided by height squared (m^2^) and BMI of <18.5, 20-24·9, 25-29·9 and ≥30 kg/m2 were considered as underweight, normal, overweight and obese, respectively^11^. The systolic and diastolic blood pressure was measured using a standard mercury sphygmomanometer three times with an interval of 30 min in participants and the average of the three measurements was taken as the blood pressure. High blood pressure was defined as BP≥140/90^12^. Serum total cholesterol, HDL, LDL and TAG, and fasting blood glucose concentrations were determined after 12 h fast. Fasting blood glucose concentrations and serum lipids were measured enzymatically using commercial kits, while serum CRP levels were determined by polyethylene glycol-enhanced immunoturbidimetry^13^.Total energy expenditure (TEE) was measured as the sum of basal energy expenditure (BEE), the energy expenditure of physical activity (EEPA) and the thermic effect of food (TEF)^14^. BEE calculated from the basic Harris-Benedict equations^15^. Overall, 10% to 20%, 25% to 40% and 45% to 60% of BEE were added for minimal, moderate and strenuous activity, respectively. TEF was measured as the 10% of BEE and EEPA.

### Assessment of Dietary Intake

Dietary information was collected using a questionnaire for 24-h dietary recall, administered by trained dietary interviewers in a face-to-face interview in Mashhad Health Centers^16,17^. This questionnaire was completed by master students of nutrition. Individual dietary intake was assessed using Dietplan6 software (Forest field Software Ltd., UK). The selected variables were carbohydrates (total carbohydrate, starch, sucrose, glucose, fructose, total sugar, maltose, lactose), total protein, fats (total fat, saturated fatty acid, MUFA, PUFA, trans fatty acid and cholesterol). Energy density was calculated by (total energy intake in day (kcal)/ weight of food intake (gr)).

### Physical activity level

Physical activity level (PAL) was evaluated using a standard questionnaire, and participants divided into 5 groups as followed: 1- extremely inactive (<1.40), 2- sedentary (1.40–1.69), 3- moderately active (1.70–1.99), 4- vigorously active (2.00–2.40), or 5-extremely active (>2.40)^18^.

### Statistical Analysis

Data were calculated using SPSS-20 software (SPSS Inc., IL, USA). Kolmogorov-Smirnov test was used to check the normality of data. Descriptive statistics including mean ±standard deviation (SD) were determined for variables with normal distribution or data were expressed as median± IQR for not normally distributed variables. For normally distributed variables, t-student test was used, while Bonferonni correction was used for multiple comparisons. The Mann-Whitney U test was used for continuous variables. For categorical parameters, Chi-square or Fisher exact tests were used. Logistic regression analysis was used to calculate association of micro/macronutrients with clinical data. All the analyses were two-sided and statistical significance was set at *P*<0.05.

## Results

### Characteristics of the population

The prevalence of underweight, overweight and obese individuals was 1.4%, 42.3%, and 30.3%, respectively. Obese group had significantly (*P*<0.05) lower physical activity level and total energy expenditure. Not surprisingly the levels of LDL, TC, hs-CRP, TG, uric acid, SBP/DBP, and glucose were significantly higher, while the HDL level was lower in the obese group, compared to the non-obese controls (*P*<0.001). Similar results were observed for the other groups compared normal weight group (Tables 1 and 2).

**Table 1:** General characteristics of the study population categorized by body mass index and derived from the Mashhad stroke and heart atherosclerotic disorders (MASHAD) study (2010-2020)

**Variables**	**Normal weight (n: 2552) **	**Underweight (n:139)**	**Overweight (n: 4154)**	**Obese (n:2964)**	** P value ^a^**	** P value ^b^**	** P value ^c^**
Gender					0.004	0.001	0.001
Female	1182	47	2370	2283
Male	1376	92	1787	678
Current smoker					0.001	0.001	0.001
No	1912	82	3328	2384
Yes	641	56	832	585
Marital status					0.491	0.065	0.001
Single	145	6	283	237
Married	2412	133	3878	2726
Education					0.192	0.003	0.001
Illiterate	350	21	497	440
Elementary	987	64	1573	1341
High school	828	38	1507	938
College	343	13	494	190
Job status					0.147	0.001	0.001
Student	2	1	14	4
Employed	1194	70	1542	721
Unemployed	1064	51	2064	1991
Retired	251	13	463	212			

^a^ Underweight versus normal weight ^b^ Overweight versus normal weight ^c^ Obese versus normal weight

**Table 2:** Clinical and biochemical characteristics of population categorized by body mass index and derived from the Mashhad stroke and heart atherosclerotic disorders (MASHAD) study (2010-2020)

** Variables**	**Normal weight**		**Underweight**		**Overweight**		** Obese **		** P value ^a^**	** P value ^b^**	** P value ^c^**
	** Mean **	**SD**	** Mean**	** SD**	** Mean**	** SD **	**Mean**	**SD **			
Age (yr)	47.9	8.5	47.6	8.1	48.5	8.2	48.4	7.9	0.410	0.009	0.001
Weight(kg)	47.9	6.5	48.2	8.6	70.8	11.8	81.6	14.8	0.001	0.001	0.001
Height (meter)	1.6	0.1	1.6	0.1	1.6	0.1	1.5	0.1	0.002	0.001	0.001
Total energy expenditure	2362.0	341.2	2380.6	384.6	2357.2	305.2	2344.0	269.9	0.740	0.326	0.015
Waist circumference (cm)	74.5	9.2	86.1	8.1	95.5	10.2	105.0	13.3	0.001	0.001	0.001
Systolic blood pressure (mmHg)	116.3	19.1	111.3	20.0	120.3	20.1	122.6	26.2	0.020	0.001	0.001
Diastolic blood pressure (mmHg)	77.3	10.4	72.5	13.5	80.5	16.6	80.0	14.6	0.011	0.001	0.001
LDL(mg/dl)	101.2	29.9	101.1	35.2	115.4	44.6	117.0	43.4	0.001	0.001	0.001
HDL(mg/dl)	46.1	11.5	43.7	15.9	41.4	12.8	41.5	12.3	0.084	0.001	0.001
Glucose(mg/dl)	80.5	16.4	77.6	15.3	83.0	20.4	85.5	22.0	0.002	0.001	0.001
Uric acid(mg/dl)	4.0	1.0	3.9	1.4	4.6	1.9	4.7	1.8	0.003	0.001	0.001
Total cholesterol (mg/dl)	166.0	33.5	185.3	38.9	189.6	50.1	193.2	51.3	0.001	0.001	0.001
Triglyceride (mg/dl)	96.9	44.4	79.5	35.4	125.5	90.3	136.5	88.1	0.001	0.001	0.001
HSCRP (mg/dl)	1.2	1.3	1.3	1.7	1.5	2.1	2.4	4.0	0.945	0.001	0.001

^a^ Underweight versus normal weight ^b^ Overweight versus normal weight ^c^ Obese versus normal weight

### Association of macronutrients intakes with obesity and Waist circumference

We then sought to investigate the relationship between macronutrient intakes in our population characterized by normal weight, underweight, overweight, obesity as well as with waist circumference. As shown in Table 3, there were significantly different levels of energy, energy density, protein, total fat, saturated fatty acid (SFA), mono-unsaturated fatty acid (MUFA), polyunsaturated fatty acid (PUFAs), trans fatty acid, cholesterol, total carbohydrate, sucrose and starch between the obese and normal weight group (*P*<0.001). These differences remained statistically significant for Trans fatty acid (*P*=0.033), lactose (*P*=0.009), fructose (*P*=0.025), glucose (*P*=0.017), sucrose (*P*=0.021) and maltose (*P*=0.015) after adjustment for energy intake. Moreover, the levels of protein, saturated Fatty acid, lactose, maltose, starch, fructose, glucose, and fiber were significantly different in subjects with high waist circumference (Table 4) (*P*<0.01).

**Table 3:** Energy and macronutrient intakes in subjects categorized by body mass index and derived from the Mashhad stroke and heart atherosclerotic disorders (MASHAD) study (2010-2020)

** Variables **	**Normal weight Mean (SD)**		**Underweight Mean (SD)**		**Overweight Mean (SD)**		**Obese Mean (SD) **		** P value ^a^**	** P value ^b^**	** P value ^c^**
	** Mean**	** SD**	** Mean**	** SD **	**Mean**	** SD **	**Mean **	**SD**			
Energy (Kcal)	1878.1	925.4	1800.5	881.5	1840.0	916.5	1736.4	837.1	0.585	0.190	0.001
Energy density	1.0	0.4	1.0	0.4	1.0	0.4	1.0	0.4	0.530	0.007	0.001
Protein(gr)
Crude	68.5	40.5	67.8	33.1	68.2	40.5	64.4	35.9	0.875	0.752	0.001
Adjusted	67.7	21.1	67.8	18.8	68.1	20.2	68.7	20.3	0.314	0.351	0.134
Fat (gr)
Crude	70.2	45.4	70.7	46.5	68.0	43.9	64.5	40.2	0.660	0.020	0.001
Adjusted	70.2	25.5	70.0	27.5	69.3	24.8	70.0	24.3	0.913	0.044	0.242
SFA (gr)
Crude	18.2	12.2	17.5	11.0	18.3	12.6	16.8	10.6	0.385	0.682	0.001
Adjusted	17.7	8.1	17.0	8.9	17.5	8.1	17.5	7.2	0.581	0.825	0.176
MUSFA (gr)
Crude	18.3	12.1	18.6	10.2	18.1	12.0	17.5	11.3	0.974	0.150	0.001
Adjusted	19.6	7.3	20.3	7.3	19.4	7.2	19.3	6.9	0.275	0.212	0.097
PUSFA (gr)
Crude	23.7	16.4	25.1	23.7	22.6	17.4	21.7	16.4	0.251	0.150	0.001
Adjusted	23.1	13.7	26.3	18.4	22.8	13.4	22.9	13.0	0.015	0.145	0.730
TFA (gr)
Crude	0.8	0.8	0.7	0.5	0.8	0.8	0.7	0.7	0.322	0.192	0.001
Adjusted	1.7	0.6	1.6	0.5	1.6	0.6	1.6	0.6	0.440	0.115	0.033
Cholesterol
Crude	195.4	214.5	174.2	214.8	191.7	215.6	175.0	209.6	0.449	0.104	0.001
Adjusted	188.0	192.3	185.4	195.7	185.7	180.0	184.0	181.7	0.963	0.74	0.445
Carbohydrate
Crude	242.6	127.0	231.4	120.1	239.7	128.5	227.0	121.8	0.385	0.490	0.001
Adjusted	237.8	65.1	231.3	64.3	242.7	62.0	239.6	62.1	0.423	0.094	0.430
Sucrose(gr)
Crude	31.4	31.0	33.3	29.7	30.6	29.2	28.1	27.9	0.947	0.203	0.001
Adjusted	30.3	27.2	33.1	28.6	29.9	26.3	28.7	24.9	0.510	0.351	0.021
Lactose(gr)
Crude	8.5	15.8	9.5	13.5	10.2	16.5	8.7	15.6	0.972	0.024	0.476
Adjusted	8.3	12.7	9.4	11.2	9.1	13.2	9.3	13.1	0.715	0.005	0.009
Maltose(gr)
Crude	2.2	2.7	1.6	2.5	2.2	2.6	2.2	2.5	0.008	0.953	0.462
Adjusted	2.7	2.3	2.6	2.2	2.8	2.3	2.9	2.3	0.033	0.281	0.015
Starch(gr)
Crude	143.5	86.2	135.8	67.4	142.3	89.4)	135.3	86.4	0.150	0.354	0.001
Adjusted	144.0	60.9	136.1	65.2	145.4	61.9	145.5	60.5	0.453	0.632	0.64
Fructose(gr)
Crude	14.4	19.5	8.45	16.7	15.5	19.1	14.3	17.6	0.015	0.125	0.814
Adjusted	15.0	16.1	10.4	15.5	15.9	17.3	15.8	15.7	0.023	0.036	0.025
Glucose(gr)
Crude	12.4	16.7	7.9	12.5	12.7	15.7	11.9	14.5	0.006	0.118	0.794
Adjusted	12.5	13.6	9.1	11.6	13.5	13.9	13.3	12.6	0.012	0.025	0.017
Fiber(gr)
Crude	15.7	11.9	14.7	11.2	15.3	12.9	15.4	12.4	0.657	0.425	0.132
Adjusted	15.7	10.8	15.8	10.9	16.1	11.2	16.1	10.7	0.825	0.313	0.145

^a^ Underweight versus normal weight ^b^ Overweight versus normal weight ^c^ Obese versus normal weight

**Table 4:** Energy and macronutrient intakes in subjects categorized by body mass index and derived from the Mashhad stroke and heart atherosclerotic disorders (MASHAD) study (2010-2020)

	**Crude energy (Kcal)**					**Adjusted energy density**				
	** Normal **		**Central obese **			**Normal**		**Central obese **		
**Variables**	** Mean**	**SD **	**Mean**	** SD**	** P value**	** Mean**	**SD **	**Mean**	**SD**	**P value**
Energy (Kcal)	1899.3	958.1	1720.1	824.7	0.001	1.0	0.4	1.0	0.4	0.009
Protein (gr)	69.8	40.4	64.3	37.1	0.001	67.7	21.1	68.6	19.9	0.004
Fat (gr)	70.9	45.4	64.6	38.7	0.001	69.6	26.1	70.1	23.6	0.237
SFA (gr)	18.8	12.4	16.7	10.9	0.001	17.7	8.2	17.4	7.3	0.007
MUSFA (gr)	18.9	12.8	16.9	11.2	0.001	19.4	7.4	19.3	6.5	0.250
PUSFA (gr)	24.2	17.5	21.4	15.8	0.001	22.6	13.9	23.1	13.5	0.144
TFA (gr)	0.8	0.8	0.7	0.7	0.001	1.6	0.6	1.6	0.6	0.699
Cholesterol (mg)	193.5	213.2	173.2	208.5	0.001	187.9	190.4	183.5	180.7	0.730
Sucrose (gr)	32.4	31.7	28.5	26.6	0.001	29.6	28.2	29.8	24.6	0.668
Lactose (gr)	8.9	15.4	8.4	15.6	0.909	8.7	12.1	9.1	12.8	0.001
Maltose (gr)	2.2	2.8	2.2	2.5	0.090	2.7	2.5	2.9	2.2	0.001
Starch (gr)	149.8	91.2	133.2	83.1	0.001	145.6	64.5	144.6	59.1	0.245
Fructose (gr)	14.9	20.1	14.3	17.4	0.004	15.1	17.5	15.9	15.8	0.035
Glucose (gr)	12.9	17.2	12.1	14.3	0.002	12.7	14.4	13.4	12.8	0.017
Fiber (gr)	15.9	13.6	15.7	12.2	0.186	15.5	11.1	16.4	10.5	0.001
Carbohydrate (gr)	250.1	135.6	224.	118.8	0.001	240.4	64.9	241.3	57.8	0.148

^a^ Nutrient intakes were adjusted for total energy intake by the residual method of linear regression Central obese: >80cm for women and >100cm for men; SFA: Saturated Fatty acid; MUSFA: Mono Unsaturated Fatty acid; PUSFA: Poly Unsaturated Fatty acid; TFA: Trans Fatty acid

The association of macronutrient intake with different categories of obesity was investigated using logistic regression model before and after adjustment based on 2 models [Model I: adjusted for age, sex and energy intake; Model II: adjusted for age, sex, energy intake, current smoking and physical activity levels] (Tables 5, 6). SFAs (*P*=0.031), PUFAs (*P*<0.001), sucrose (*P*<0.001) and starch (*P*= 0.045) were related to obesity, while in model 2, this association remained only for sucrose (*P*<0.001). A significant relationship was detected for fat in model 1 and 2 in the overweight group, compared to normal weight subjects (*P*=0.034 and *P*=0.031, respectively).

**Table 5:** Association of macronutrient intakes of protein, fat & energy with obesity compared to normal weight P value

**Variables **	**Underweight** ** Odds ratio (95% CI)**	**P value**	**Overweight ** **Odds ratio (95% CI) **	**P value **	**Obese** **Odds ratio (95% CI)**	**P value **
Energy density
Crude	1.52 (0.86, 2.62)	0.145	1.28 (1.09, 1.51)	0.003	1.49 (1.26, 1.78)	0.001
Model I	1.57 (0.91, 2.71)	0.137	1.26 (1.06, 1.48)	0.006	1.31 (1.09, 1.57)	0.003
Model II	1.56 (0.95, 2.61)	0.125	1.17 (0.98, 1.39)	0.070	1.13 (0.91, 1.41)	0.250
Protein (gr)
Crude	1.00 (0.99, 1.01)	0.765	1.00 (0.99, 1.00)	0.981	0.99 (0.99, 1.00)	0.001
Model I	1.00 (0.99, 1.01)	0.117	1.00 (0.99, 1.00)	0.135	1.00 (0.99, 1.00)	0.133
Model II	1.00 (0.99, 1.01)	0.007	1.00 (0.99, 1.00)	0.262	1.00 (0.99, 1.00)	0.516
Fat(gr)
Crude	1.00 (0.99, 1.01)	0.558	0.99 (0.99, 1.00)	0.032	0.99 (0.99, 1.00)	0.001
Model I	1.00 (0.99, 1.01)	0.933	0.99 (0.99, 1.00)	0.034	0.99 (0.99, 1.00)	0.067
Model II	1.00 (0.99, 1.01)	0.731	0.99 (0.99, 1.00)	0.031	0.99 (0.99, 1.00)	0.772
SFA (gr)
Crude	0.98 (0.96, 1.01)	0.265	0.99 (0.99, 1.00)	0.657	0.98 (0.97, 0.98)	0.001
Model I	0.99 (0.95, 1.02)	0.663	0.99 (0.99, 1.00)	0.874	0.98 (0.97, 0.99)	0.031
Model II	0.98 (0.95, 1.02)	0.534	1.00 (0.99, 1.01)	0.853	1.00 (0.99, 1.01)	0.714
MUFA (gr)
Crude	0.99 (0.97, 1.02)	0.978	0.99 (0.99, 1.00)	0.181	0.98 (0.97, 0.99)	0.001
Model I	1.01 (0.98, 1.05)	0.244	0.99 (0.98, 1.00)	0.215	0.99 (0.98, 1.00)	0.142
Model II	1.02 (0.98, 1.05)	0.245	0.99 (0.98, 1.00)	0.267	1.00 (0.99, 1.01)	0.585
PUFA (gr)
Crude	1.00 (0.99, 1.02)	0.223	0.99 (0.99, 1.00)	0.352	0.99 (0.98, 0.99)	0.001
Model I	1.02 (1.00, 1.03)	0.008	0.99 (0.99, 1.00)	0.223	1.01 (1.00, 1.02)	0.001
Model II	1.02 (1.00, 1.04)	0.003	0.99 (0.99, 1.00)	0.167	0.99 (0.99, 1.00)	0.520
TFA (gr)
Crude	0.76 (0.51, 1.13)	0.171	0.94 (0.87, 1.02)	0.191	0.81 (0.74, 0.89)	0.001
Model I	0.85 (0.57, 1.27)	0.442	0.94 (0.86, 1.03)	0.224	0.89 (0.80, 1.00)	0.050
Model II	0.78 (0.51, 1.19)	0.264	0.96 (0.87, 1.05)	0.428	1.00 (0.88, 1.15)	0.932
Cholesterol (mg)
Crude	1.00 (0.99, 1.00)	0.782	1.00 (0.99, 1.00)	0.321	0.99 (0.99, 1.00)	0.001
Model I	1.00 (0.99, 1.00)	0.911	1.00 (0.99, 1.00)	0.923	1.00 (0.99, 1.00)	0.861
Model II	1.00 (0.99, 1.00)	0.946	1.00 (0.99, 1.00)	0.865	1.00 (0.99, 1.00)	0.344

**Table 6:** Association of macronutrient intakes of carbohydrate with obesity compared to normal weight

**Variables **	**Underweight ** **Odds ratio (95% CI)**	**P value**	**Overweight** ** Odds ratio (95% CI) **	**P value **	**Obese** ** Odds ratio (95% CI)**	**P value **
Carbohydrate (gr)
Crude	1.00 (0.99, 1.00)	0.245	1.00 (0.99, 1.00)	0.655	0.99 (0.99, 1.00)	0.001
Model I	1.00 (0.99, 1.01)	0.313	1.00 (0.99, 1.01)	0.142	1.00 (0.99, 1.01)	0.212
Model II	0.99 (0.99, 1.00)	0.180	1.00 (0.99, 1.01)	0.121	1.00 (0.99, 1.00)	0.965
Sucrose (gr)
Crude	1.00 (0.99, 1.00)	0.994	0.99 (0.99, 1.00)	0.136	0.99 (0.99, 1.00)	0.001
Model I	1.00 (0.99, 1.01)	0.543	0.99 (0.99, 1.00)	0.236	0.99 (0.99, 1.00)	0.006
Model II	1.00 (0.99, 1.00)	0.561	0.99 (0.99, 1.00)	0.275	0.99 (0.99, 1.00)	0.013
Lactose (gr)
Crude	1.00 (0.99, 1.01)	0.475	1.00 (0.99, 1.00)	0.281	1.00 (0.99, 1.00)	0.931
Model I	1.00 (0.99, 1.01)	0.283	1.00 (0.99, 1.00)	0.280	1.00 (0.99, 1.00)	0.840
Model II	1.00 (0.99, 1.01)	0.577	1.00 (0.99, 1.00)	0.220	1.00 (0.99, 1.00)	0.994
Maltose (gr)
Crude	0.85 (0.75, 0.96)	0.014	0.99 (0.97, 1.01)	0.604	0.97 (0.95, 1.00)	0.062
Model I	0.89 (0.79, 0.99)	0.034	1.00 (0.98, 1.02)	0.797	1.01 (0.98, 1.03)	0.393
Model II	0.86 (0.77, 0.97)	0.015	1.00 (0.98, 1.03)	0.714	1.00 (0.96, 1.03)	0.101
Starch (gr)
Crude	0.99 (0.99, 1.00)	0.051	1.00 (0.99, 1.00)	0.480	0.99 (0.99, 1.00)	0.001
Model I	1.00 (0.99, 1.00)	0.369	1.00 (0.99, 1.00)	0.514	1.00 (0.99, 1.01)	0.045
Model II	0.99 (0.99, 1.00)	0.124	1.00 (0.99, 1.01)	0.191	1.00 (0.99, 1.01)	0.071
Fructose (gr)
Crude	0.97 (0.96-0.99)	0.015	1.00 (0.99, 1.00)	0.362	0.99 (0.99, 1.00)	0.111
Model I	0.98 (0.96-1.00)	0.051	1.00 (0.99, 1.01)	0.144	1.00 (0.99, 1.00)	0.963
Model II	0.98 (0.97-1.00)	0.173	1.00 (0.99, 1.00)	0.645	0.99 (0.99, 1.00)	0.191
Glucose (gr)
Crude	0.97 (0.95,0.99)	0.015	1.00 (0.99, 1.00)	0.451	0.99 (0.99, 1.00)	0.113
Model I	0.98 (0.96,1.00)	0.045	1.00 (0.99, 1.00)	0.243	1.00 (0.99, 1.00)	0.876
Model II	0.98 (0.96,1.00)	0.138	1.00 (0.99, 1.00)	0.566	0.99 (0.99, 1.00)	0.431
Fiber(gr)
Crude	0.98 (0.96,1.01)	0.342	1.00 (0.99, 1.00)	0.901	0.99 (0.98, 0.99)	0.020
Model I	1.00 (0.97,1.02)	0.958	1.00 (0.99, 1.00)	0.953	0.99 (0.98, 1.00)	0.325
Model II	1.00 (0.97,1.02)	0.976	1.00 (0.99, 1.00)	0.941	0.99 (0.98, 1.00)	0.15

## Discussion

To the best of our knowledge, this study is the first to explore the impact of macronutrients intake in a large population containing 9809 subjects divided into 4 groups, normal weight, and overweight, underweight and obese individuals as well as with respect to central obesity. Our findings demonstrate the association of Trans fatty acids, lactose, fructose, glucose, sucrose, and maltose, after adjustment for energy intake, with obesity and adiposity. Additionally, this association was also observed for lactose, fructose, and glucose in overweight group, compared to normal weight group, suggesting the important role of energy intake for increasing BMI, categorized by adiposity. In this regard, public health experts believe that dietary change is effective in the prevention and treatment of obesity^19,20^.

Food intake of Iranian population is 40% higher than required amount (40% more carbohydrates and 30% more fats) ^21^ and similar results are reported in Malaysian study ^22^. Carbohydrate, protein, and fat are the major sources of energy, and their excess consumption will lead to a positive energy balance. Our data suggest that there was no significant difference in total carbohydrate, protein and fat intake between normal weight, overweight and obese individuals from Iran. Furthermore, energy intake in normal weight was higher than overweight and obese individuals. Hence weight differences are likely to be due to the increase in energy expenditure such as physical activity and not energy intake, which is in agreement with other studies^23,24^. However, various factors are related to obesity such as genetic, environmental (dietary nutrient intake, smoking) and metabolic factors^25,20^. Moreover, the results of National Health and Nutrition Examination Survey in the USA showed that the replacement of dietary fat with dietary carbohydrate did not alter the incidence of obesity in the population^26^. High total energy intake is usually related to a high total sugar intake while several other studies revealed inverse relationship between sugar intake and BMI^26-31^. In line with these observations, our data showed an association of lactose, fructose, glucose, sucrose, and maltose after adjustment for energy intake with respect to obesity. BMI is related to daily sugar intake, but no significant relationship with total calories, protein, fat or carbohydrates intake^32^. On the other hand, there is increasing evidence showing the association of protein intake and BMI^33,34^. However, a lack of this relationship was showed with BMI^35,36^ which are in agreement with our data.

We evaluated the correlation between fat consumption with weight. We observed the significant reduction of monosaturated fatty acid and Trans fatty acid in obese subjects after adjustment with energy intake. Several studies have been shown a positive association between fat intake and obesity^38^ although this is not a consistent finding^38,39^. The low incidence of obesity was reported in an Eskimo population with a high-fat diet in their diet^40^. On the other hand, another study has reported the association of obesity with consuming oil-rich diets in some Arabic countries including United Arab Emirates, Saudi Arabia and Kuwait ^41^.Total fat has a relation to BMI while these relations were inverse for monounsaturated fat and polyunsaturated fat^42^. This conflicting data supports the need for further investigation of the role of fat consumption with obesity.

A major strength of the present study was that it was carried out in a large number, while the main limitation is age and gender differences between groups. Another limitation was using 24-h dietary recall because it cannot cover all dietary intake (weekly, monthly and yearly) although these variables were adjusted in logistic regression model.

## Conclusion

Various genetic and environmental factors are related to obesity. On the other hand, environmental factors like dietary nutrient intake play an important role in the progression of the obesity. We demonstrated the association of fatty acid, lactose, fructose, glucose, sucrose, and maltose with obesity after adjustment for energy intake, suggesting the important role of sugar with body mass index. Further studies are warranted to investigate the association of carbohydrate, protein and fat intake with obesity.

## Conflict of interest statement

The authors have no conflict of interest to disclose.

## Funding

This work was supported by grant from in Mashhad University of Medical Sciences.

## Highlights

- Obese subjects had a higher serum LDL-cholesterol, total cholesterol, triglycerides, and glucose compared to normal group.
- Obese subjects had high levels of serum hs-CRP, uric acid, and blood pressures compared to normal group.
- There was a significant difference in the dietary intakes of the groups categorized by BMI.
- Obese subjects had high dietary intakes of protein, fat & carbohydrates.
